
JOURNAL INFORMATION
==============================
NLM Title Abbreviation: Insights Imaging
Journal ID: Insights Imaging
NLM Journal ID: 101532453

Insights into Imaging

EISSN: 1869-4101
Publisher: Springer

ARTICLE INFORMATION
==============================
PMCID: PMC8885130
PMID: 35229236
DOI: 10.1186/s13244-022-01168-w
Article ID: 1168
Article version: 2
Subjects: Meeting Abstracts

EuSoMII Virtual Annual Meeting 2021 ‘Connections’ Book of Abstracts

Electronic publication date: 2022 Mar 1
Publication date: 2022 Mar
Volume: 13
Issue: Suppl 1
Issue sponsor: EuSoMII 2021 is the annual meeting of the European Society of Medical Imaging Informatics. Publication costs were not supported by sponsorship.
Electronic Location ID: 31
Copyright: © The Author(s) 2022
License: Open AccessThis article is licensed under a Creative Commons Attribution 4.0 International License, which permits use, sharing, adaptation, distribution and reproduction in any medium or format, as long as you give appropriate credit to the original author(s) and the source, provide a link to the Creative Commons licence, and indicate if changes were made. The images or other third party material in this article are included in the article's Creative Commons licence, unless indicated otherwise in a credit line to the material. If material is not included in the article's Creative Commons licence and your intended use is not permitted by statutory regulation or exceeds the permitted use, you will need to obtain permission directly from the copyright holder. To view a copy of this licence, visit http://creativecommons.org/licenses/by/4.0/.
License URL: https://creativecommons.org/licenses/by/4.0/

Conference name: EuSoMII Virtual Annual Meeting 2021 ‘Connections’
Conference location: Vienna, Austria
Conference date: 23 October 2021
issue-copyright-statement: © The Author(s) 2022

==============================

### Insights into Imaging

Education and strategies in European radiology

EuSoMII Virtual Annual Meeting 2021 'Connections' Book of abstracts / Volume 13 / Supplement 1

EuSoMII VirtualAnnual Meeting 2021

Connections

Wien, 23rd October 2021

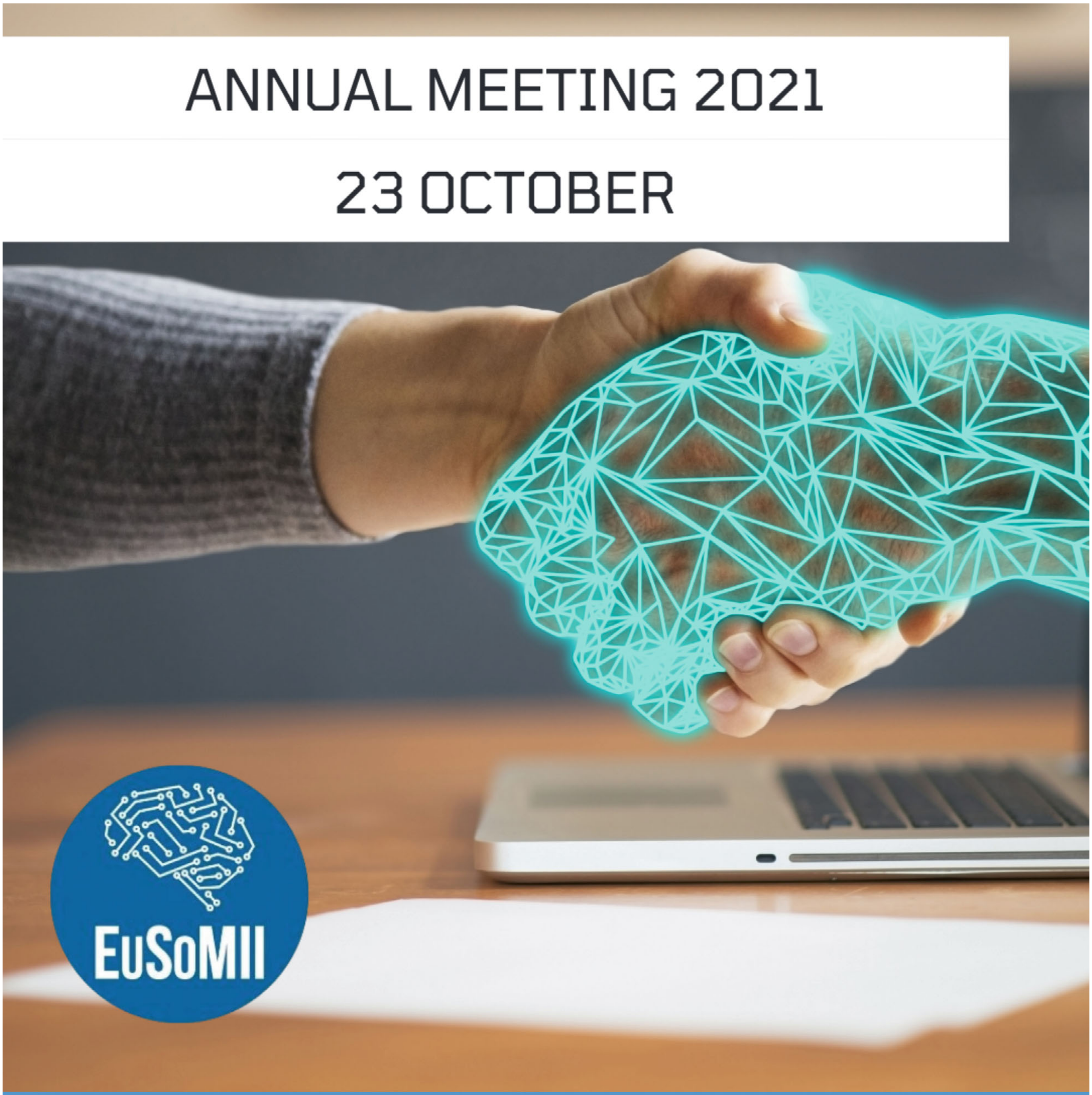

### EuSoMII Virtual Annual Meeting 2021
### 'Connections'
### Book of Abstracts

EuSoMII European Society of Medical Imaging Informatics
Am Gestade 1, 1010, Vienna - info@eusomii.org - www.eusomii.org

### SS 1 - AWARDED ABSTRACT 'GOLD MEDAL'

### Impact of deep learning reconstruction and CT dose on automatic lung vessel morphology characterization software: a 3D-printed anthropomorphic phantom study

*I.Hernandez-Giron[1], Z. Zhai[2], W.J.H.Veldkamp[3], J.M. den Harder[2], B. Stoel[1]*

*[1]Division of Image Processing (LKEB), Radiology Department, Leiden University Medical Center (LUMC), The Netherlands*
*[2]Amsterdam University Medical Center (AMC), The Netherlands*
*[3]Radiology Department, Leiden University Medical Center (LUMC), The Netherlands*

Short Summary: Automated methods for disease detection and characterization are becoming widely used to alleviate radiologists' workload. Radiologic images are adapted to human visual perception. The influence of acquisition/reconstruction on image quality and automatic diagnostic tools performance needs to be investigated to allow generalizability (protocols, systems and manufacturers). Advanced image reconstruction in Computed Tomography (iterative and AI-based), rely on patient morphometry and anatomy. An anthropomorphic 3D-printed lung vessel phantom, as a patient surrogate, was used to test CT dose and reconstruction influence on the performance of an automated method for vessel quantification. Vessel detection improved with increasing dose for all reconstruction methods. With deep learning-based reconstruction more vessels were accurately detected and classified.

Purpose/Objectives: To evaluate the influence of dose and reconstruction on the performance of an automated vessel extraction and classification algorithm for CT images of an anthropomorphic phantom.

Methods and materials: A 3D-printed lung vessel phantom (material Visijet-EX200; 0.1-4.25mm radius range) inside a PMMA thorax-shaped holder was scanned [CT-thorax protocol; (CTDIvol=4.0-2.1-1.0-0.5-0.2mGy); Canon_Aquilion_Prism, 4 repetitions]. Images were reconstructed with filtered-back-projection (FBP-FC08), iterative (AIDR3De-FC08) and deep-learning (DL) (AiCE-lung-standard) methods.

An automated in-house graph-cuts-based method for pulmonary vessel extraction and quantification, measured on the images, for each radius, the median pixel value (MPV, Hounsfield units-HU) and inter-quartile-range of pixel values (IQR, noise measurement) together with the total volume of voxels identified as vessels (averaged over 4 acquisitions).

Results: As an example, for 3mm-radius, (MVP±σ) were, for (CTDIvol=0.2-0.5-1.0-2.1-4.0 mGy): [FBP: (98±5HU)-(97±9HU)-(93±7HU)-(94±7HU)-(91±2HU)]; [iterative: (101±3HU)-(104±4HU)-(104±7HU)-(102±4HU)-(99±3HU)]; [DL-based: (106±5HU)-(102±10HU)-(106±8HU)-(107±11HU)-(103±6HU)].

The IQR decreased with increasing dose for all reconstructions: (IQR±σ; 3mm-radius) were, for (CTDIvol=0.2-0.5-1.0-2.1-4.0 mGy): [FBP: (120±6HU)-(72±5HU)-(44±3HU)-(34±4HU)-(37±10HU)]; [iterative: (62±8HU)-(42±2HU)-(33±9HU)-(27±4HU)-(25±4HU)]; [DL-based: (62±12HU)-(59±8HU)-(43±7HU)-(39±5HU)-(36±6HU)].

The average detected vessel tree volume (ml) varied with dose and reconstruction: [FBP: (7.14±0.02ml)-(5.66±0.03ml)-(5.39±0.01ml)-(5.33±0.05ml)-(5.24±0.07ml)]; [iterative: (4.22±0.06ml)-(4.91±0.10ml)-(5.19±0.04ml)-(5.41±0.08ml)-(5.47±0.05ml)]; [DL-based: (6.36±0.07ml)-(7.15±0.08ml)-(7.22±0.06ml)-(7.39±0.03ml)-(7.42±0.03ml)].

Conclusion: Reconstruction method and dose affected vessel detection output (more vessels detected with DL-reconstruction and with increasing dose). 3D-printed anthropomorphic phantoms with known structures are useful to test objectively the performance of automated tools for clinical diagnosis.

Disclosure: Veni personal grant to I Hernandez-Giron (Pr.Nr.17378) funded by NWO: Through the eyes of AI-safe and optimal integration of Artificial Intelligence in Radiology.

Phantom creation: CLUES project (NWO Pr.Nr.13592)

Keywords: 3D printing, image quality, phantom, CT, automatic vessel detection, deep learning image reconstruction

Insights Imaging (2022) 13 (Suppl 1): 31
https://doi.org/10.1186/s13244-022-01168-w
Published: 01 March 2022

### SS 2

### The Unifesp Radiology Report Dataset

*Eduardo M. Farina, MD; Murilo M. de Freitas, MD; Nitamar Abdala, MD, PhD; Marcelo O. Coelho, MD; Errol Colak MD, FRCPC, HBSc; Igor Santos, MD; Suely F. Ferraciolli, MD; Felipe C. Kitamura, MD, PhD*

Short Summary: We present a Brazilian Portuguese Radiology Report Dataset annotated for critical findings from a public institution.

Purpose/Objectives: To develop an open radiology report dataset in Brazilian Portuguese annotated for critical findings.

Methods and materials: The construction of the dataset was done by extracting every CT scan radiology report from 2014-2021. We performed automatic anonymization using Regex to remove patient and physician names, identification numbers, and dates. The second step of anonymization was listing unique words and performing a manual replacement of them in the reports. The last step was during the annotation process we searched for any remaining identification in the report that was not removed by our automated process and we did manual removal.

Results: The first version of the dataset comprises 557 de-identified radiology reports of CT scans from different body parts and annotations for critical findings (74 positives, 483 negatives). The dataset is available at https://github.com/DDI-UNIFESP-AI-Informatics-in-Radiology/UNIFESP-Radiology-Report-Dataset and will be constantly updated.

Conclusion: We developed a Brazilian Portuguese radiology report dataset annotated for critical findings.

Disclosure: There is no conflict of interest to declare.

Keywords: Radiology reports; dataset; open-science;

### SS 3

### Deep learning for salivary gland tumours segmentation and classification based on CT images.

*Lorenzo Ugga; Gaia Spadarella; Serena D'Aniello; Vincenzo Abbate; Giovanni Dell'Aversana Orabona; Edoardo Prezioso; Stefano Izzo; Fabio Giampaolo; Luigi Califano; Renato Cuocolo; Francesco Piccialli*

Short Summary: Deep learning model for segmentation and classification of salivary gland tumours has proven promising, potentially improving patient management.

Purpose/Objectives: This study aims to develop and evaluate a deep learning network for characterizing salivary gland tumours, based on non-contrast CT images.

Methods and materials: Pre-operative CT volumes of patients affected by salivary gland tumour were retrospectively analyzed. CT examinations were obtained on different scanners (16- or 64-slice) with variable acquisition parameters (slice thickness: 0.5-2 mm; in-plane resolution: 0.5-1 mm). Soft tissue reconstruction algorithm volumes before contrast agent administration were selected for the analysis. Lesions were identified by two radiologists experienced in head and neck imaging who subsequently proceeded to manual lesion 3D segmentation. Tumor class was in all cases histopathologically determined.

Regarding image pre-processing, resampling to 2×2×2 mm3 was applied, density values were clipped within [−400, 400] and then scaled between 0 and 1 with a linear min-max operation. Given the limited number of patients and the complexity of the DL models to train, data augmentation was performed on the train set using different strategies including small rotation, large rotation, translation, flipping, scaling, and elastic deformation. A modified V- Net model was employed for the lesion 3D segmentation task on the tensors. Then, Residual Network 50, a convolutional network composed of 50 layers, was trained to classify benign and malignant lesions as selected 2D slices of the region. The Dice similarity coefficient, the Quantile Hausdorff Distance and the Average Hausdorff Distance were calculated to compare the results of the automatic segmentation with the ground truth provided by radiologists. Differently, evaluation metrics for the classification task included Accuracy, Precision, Recall, Specificity, and F1-score.

Finally, a per-epoch learning process analysis was carried out to increase the explainable transparency of our framework predictions.

Results: A total of 88 lesions were included.

The training and test sets consisted of 61 and 27 cases, respectively. Regarding the segmentation step, our methodology obtained on the test set the Dice score 0.85, and the 95% quantile-Hausdorff distance 4.6 on average. For the final step of the classification, the obtained accuracy was 0.89 and the F1-score 0.88 on average.

Conclusion: The proposed model has proven promising for salivary gland tumour diagnosis, suggesting both the position and the type of the lesion. It may potentially improve patient management and surgical strategy making a more accurate preoperative lesion classification.

Disclosure: A paper based on this study has been published after the abstract presentation at the EuSoMII Annual Meeting 2021 (DOI: 10.1109/JBHI.2021.3120178).

Keywords: salivary gland tumours; diagnostic imaging; CT; artificial intelligence; deep learning

### SS 4

### Privacy-preserving training of deep neural networks in large scale medical infrastructures

*Erfan Darzidehkalani, P.M.A van Ooijen*

Short Summary: Aggregation of medical image data helps to build accurate deep learning models. However, this is not always feasible due to strict data protection regulations. Federated Learning (FL) is a new technology that enables researchers to build large networks and share trained models without jeopardizing patient personal data. Federated Learning is an evolving and growing technology that provides educational institutions with secure access to data. This facilitates global collaboration and will redefine the AI paradigm in radiology in the near future.

Purpose/Objectives: In this manuscript, we introduce the FL concept to medical imaging society, and discuss its critical role in privding the environment for large-scale collaboration of medical institutions.

Methods and materials: The main FL methods are FedAvg, Single Weight Transfer (SWT), and Cyclic Weight Transfer (CWT). In FedAvg, local models are trained at each hospital and models are averaged round by round from a central server. In SWT, the model goes through the institution only once, and the global model is updated as it goes through each client. CWT is similar to SWT, only the model goes through the hospital cyclically and multiple times.

Results: FL has shown great promise in several areas of radiology as existing literature suggests. FL has been successfully deployed in COVID-19 research, Lung nodule detection, retinotherapy, mammography,breast cancer detection,MR image reconstruction , brain tumor segmentation , brain tumor type classification, and patient similarity analysis. With preserving patients private information and without revealing sensitive data.

Data-related issues, such as heterogeneous data profiles and low-quality clients, affect FL network performance. Potential solutions are FAIR data collection, data standardization, and bias-reducing algorithms.

Security and privacy are also other important issues. Patient Re-identification, sensitive data retrieval, and adversarial attacks are the most important threats to an FL network. Countermeasures like model encryption, differential privacy (DP), and data perturbation are popular measures to protect private data.

Conclusion: FL and AI are growing fields and are expected to gain more trust from medical experts and open their way to more medical centers. Technologies like Natural Language Processing (NLP) are vital to extracting information from other data types in addition to the imaging data. A large pool of institutions with various data types opens the way to use real-time big data technologies in FL networks.

Disclosure: The authors declare that there is no conflict of interest.

Keywords: Federated learning, Medical image processing, privacy-preserving deep learning

### SS 5

### Prediction of Antidepressant Treatment Response Using Machine Learning For Neuroimaging

*Farzana Z. Ali,[1] MD, MPH; Ramin Parsey,[2] MD, PhD; Christine DeLorenzo,[1,2] PhD*

*Department of [1]Biomedical Engineering and [2]Psychiatry, Stony Brook University, Stony Brook, NY, USA*

Short Summary: A newly developed machine learning algorithm shows potential for predicting remission (absence of depression) following antidepressant treatment using brain MRI acquired before initiating treatment.

Purpose/Objectives: Develop a machine learning algorithm using pretreatment brain structural MRI (sMRI) data to predict final antidepressant response after eight weeks of treatment.

Methods and materials: This study used pretreatment sMRI from a multi-site clinical trial on participants with depression (n=177) who were initiating treatment. For each individual MRI scan, 468 imaging measures including average and standard deviation of cortical thickness (mm) and gray matter volume ($mm^3$) of brain regions were automatically derived using the Freesurfer software at a single site. The imaging measures, along with the participants' age, sex, scan site, treatment assignment (placebo or selective serotonin reuptake inhibitor (SSRI)) and handedness (measured using Edinburgh Handedness Inventory (EHI) 20-item questionnaire) information, were partitioned into 60% training, 20% cross-validation and 20% test sets to avoid data leakage. A reduced number of imaging features were selected using Pearson's correlation to remove highly correlated features, and Recursive Feature Elimination with Cross-Validation. The selected features were entered into a tree boosting classifier called XGBoost to predict remission after eight weeks, following optimization of model hyperparameters.

Results: Our predictive model showed 72.22% accuracy with 54% sensitivity and 83% specificity for predicting remission in antidepressant treatment. The XGBoost model ranked 10 most predictive neuroimaging features for antidepressant efficacy, and the average cortical thickness of left opercular part of inferior frontal gyrus (posterior part of Broca's area) was the most predictive feature. This region has previously shown higher functional connectivity in depression, that lowers with medication, and may relate to the motor-related slowing, fatigue and reduced energy symptoms associated with depression.

Conclusion: This study pioneers the application of tree boosting classifier for developing a predictive algorithm for antidepressant response using neuroimaging data. The machine learning techniques applied in this research will provide valuable guidance for use of high dimensional, small sample neuroimaging data within predictive algorithms. Our future research will focus on improving accuracy and sensitivity by modifying hyperparameters of the current model for clinical utility.

Disclosure: Dr. Ali, Dr. Parsey, and Dr. DeLorenzo declare that they have no relevant or material financial interests that relate to the research described in this paper.

Keywords: MRI, prediction algorithm, depression, SSRI, XGBoost

### SS 6

### Deep learning for classification of musculoskeletal x-ray images.

*H. P. Tran, A. Fink, E. Kellner, M. Reisert, E. Kotter, F. Bamberg, M. Russe*

Short Summary: An automated AI-based classification of radiographs into predefined body regions and projections will enhance clinical workflows and more specialized region-specific networks can be used. We developed an AI algorithm with an excellent performance in classifying MSK radiographs

Purpose/Objectives: Developing a robust algorithm for classification of musculoskeletal radiographs in the most common projections of predefined body regions.

Methods and materials: Musculoskeletal radiographs from our department from 2018-2019 were classified into 15 predefined body parts and 30 projections. 14100 images were annotated on our scientific medical imaging platform Nora and exported for the deep learning study (9492 images for the training dataset, 4108 for validation, 500 images for network testing). Inception-v3, an established convolutional neural network by C. Szegedy et al., was modified with Tensorflow 2.4, developing a deep learning model with a custom network-top on a fully retrainable base model. Images were rescaled to 256*256 pixels and as a 3-level image (image, inverted image, edge-optimized image). Data was randomized, balanced, and mildly augmented. The amount of training epochs was set to 200, using a batch size of 50 with 100 steps of batching per epoch. Learning rate was reduced from 0.1 to 0.05. Training was performed with a standard graphics unit (Nvidia Tesla K80). Calculation and visualization of the results used scikit-learn and tf-explain with implementation of Gradient-weighted Class Activation Mapping.

Results: CNN training took 2:32h. Image processing of all 500 test-images took 31sec. The overall accuracy of the separate test sample was 97,6%. The f1-score of each class ranged from 0.67 to 1,00. Rare body projections were in the lower range, e.g. the hip AP view with 0,67. Lager classes like the knee AP view and the knee lateral view achieved an excellent result each of 1,00 and 0,97. However, classes with unique anatomical appearance could show good results even with reduced numbers of cases. Noticeable errors were shown between the groups of the forefoot oblique view to the foot oblique view, or the hip AP view to the Lauenstein view of the hip where the overlap in clinical routine is often also present.

Conclusion: The algorithm demonstrated an excellent classification rate of MSK radiographs in the most common projections. Classification of radiographs into predefined body regions and projection using the presented approach will enable an automated use of AI-based algorithms with more specialized region-specific networks in clinical workflow.

Disclosure: None.

Keywords: musculoskeletal radiographs, classification, AI

### SS 7

### Importing and serving open-data medical images to support Artificial Intelligence research

*Sébastien Jodogne (ICTEAM, UCLouvain, Louvain-la-Neuve, Belgium)*

Short Summary: The training and validation of Artificial Intelligence models require large volumes of high-quality data that is relevant to clinical practice. The data collection and the labeling of such images is a hard, expensive process. In the field of oncology, this need for databases of clinical images shared by multiple research teams led to the creation of The Cancer Imaging Archive (TCIA) initiative. TCIA gathers many collections of real-world images of cancers, acquired under multiple imaging modalities, that are de-identified and publicly accessible as open data. We developed an easy-to-use and intuitive interface to import images from TCIA to an open PACS ecosystem.

Purpose/Objectives: Research in Artificial Intelligence for medical imaging requires large volumes of high-quality, labeled data. The Cancer Imaging Archive (TCIA) is a public repository of DICOM images related to oncology. The aim of this work is to provide researchers and developers with a simple way to import images from the TCIA servers onto a local PACS environment.

Methods and materials: TCIA provides an application programming interface (REST API) that enables third-party applications to access the content of its collections. Orthanc is an open-source DICOM server that can be deployed by research teams as their PACS server. The deliverable of this work is an original, open-source plugin for Orthanc that imports images from TCIA using its REST API.

Results: The developed plugin takes the form of an easy-to-use Web application to browse TCIA collections and import their images. The imported images are served according to the DICOM standard, and can be immediately displayed using zero-footprint viewers.

Conclusion: This work proposes a solution to import real-world, open-data medical images into an open environment that is similar to clinical setups, which is essential to Artificial Intelligence research. Those images come from TCIA, that contains multiple acquisitions of various body parts acquired by different modalities, making it useful in many fields of radiology. Future work will take advantage of the developed plugin in research projects related to AI applied to oncology. The connection of Orthanc to other collections of open-data DICOM images will also be investigated.

Disclosure: Sébastien Jodogne is shareholder of Osimis SA.

Keywords: Open-data, Open-source, DICOM, Machine learning

### SS 8

### Automatically publishing medical images from a filesystem as a DICOM server

*Sébastien Jodogne* (ICTEAM, UCLouvain, Louvain-la-Neuve, Belgium)

Short Summary: In the context of a research team, the DICOM files associated with the subjects of some clinical study are typically stored within a hierarchy of folders located on one large network filesystem that is shared between the researchers. Such folders often contain a flat set of multiple DICOM files. It is a tedious, error-prone task to make a sensible organization of such a filesystem by hand. We developed an open-source software solution that transparently indexes all the DICOM instances that can be found on some filesystem, and that automatically publishes these resources according to the DICOM standard.

Purpose/Objectives: Research in medical imaging necessitates rigorous management of the image databases. Typical clinical trials and the training of Artificial Intelligence algorithms require to manage thousands of DICOM instances. The aim of this work is to provide researchers with a standardized way to transparently, rapidly index the content of a filesystem containing large amounts of heterogeneous imaging data.

Methods and materials: The DICOM standard specifies the well-known patient/study/series/instance hierarchy as a model of the real world. Orthanc is an open-source DICOM server that can be deployed by research teams as their PACS server. We introduce a strategy to use Orthanc as a platform that publishes the content of a filesystem according to the DICOM model of the real world.

Results: The deliverable of this work is an original, open-source plugin for Orthanc that continuously synchronizes the content of an Orthanc server with the content of a filesystem. This way, the filesystem is automatically organized according to the DICOM model of the real world, without any manual intervention. The indexed DICOM resources are immediately available in a Web interface and in a Web viewer, and can be queried/retrieved by DICOM clients.

Conclusion: This work proposes a simple, automated method to seamlessly and effectively organize a filesystem containing medical images in a standardized way, by publishing them like a DICOM server would. Future work will take advantage of the developed plugin in research projects related to Artificial Intelligence applied to oncology.

Disclosure: Sébastien Jodogne is shareholder of Osimis SA.

Keywords: Open-source, DICOM, Imaging databases

### SS 9

### Artificial intelligence: guidance for clinical imaging and therapeutic radiography workforce professionals , a Society and College of Radiographers publication

*Malamateniou Christina, Tracy O'Regan and the AI working group of the SCoR*

Short Summary: Artificial intelligence (AI) has started to be increasingly adopted in medical imaging and radiotherapy clinical practice, however research, education and partnerships have not really caught up yet to facilitate a safe and effective transition. This review offers the most up-to-date recommendations for clinical practitioners, researchers, academics and service users of clinical imaging and therapeutic radiography services. Radiography practice, education and research must gradually adjust to AI-enabled healthcare systems to ensure gains of AI technologies are maximised and challenges and risks are minimised.

Purpose/Objectives: The aim is to provide baseline guidance for radiographers working in the field of AI in education, research, clinical practice and stakeholder partnerships. The guideline is intended for use by the multi-professional clinical imaging and radiotherapy teams, including all staff, volunteers, students and learners.

Methods and materials: The recommendations have been subject to a rapid period of peer, professional and patient assessment and review. Feedback was sought from a range of SoR members and advisory groups, as well as from the SoR director of professional policy, as well as from external experts. Amendments were then made in line with feedback received and a final consensus was reached.

Results: AI is an innovative tool radiographers will need to engage with to ensure a safe and efficient clinical service in imaging and radiotherapy. Educational provisions will need to be proportionately adjusted by Higher Education Institutions (HEIs) to offer the necessary knowledge, skills and competences for diagnostic and therapeutic radiographers in a digitally-enabled future. Radiography-led research in AI should address key clinical challenges and enable radiographers to co-design, implement and validate AI solutions. Partnerships are key in ensuring the contribution of radiographers is integrated into healthcare AI ecosystems for the benefit of the patients and service users

Conclusion: Radiography is starting to work towards a future with AI-enabled healthcare. This guidance offers some recommendations for different areas of radiography practice. There is a need to update our educational curricula, rethink our research priorities, forge new strong clinical-academic-industry partnerships to optimise clinical practice. These recommendations aim to serve as baseline guidance for UK radiographers and, given the fast-changing pace of AI in healthcare, they need to be regularly updated for currency and relevance.

Disclosure: Nothing to disclose

Keywords: artificial intelligence; adoption; guidance; training; radiography

### SS 10

### Development of an AI-based model for Chest X-ray quality assessment

*Rémi Khansa, Gabriel Misrachi, Marie-pierre Revel, Guillaume Chassagnon, Souhail Bennani*

Short Summary: We conducted a deep learning-based model to detect quality deficient Chest X-Rays.

Purpose/Objectives: Chest radiography is the first-line imaging modality for diagnosing thoracic pathologies. Diagnostic accuracy may be drastically reduced due to technical limitations resulting in poor image quality and thus leading to incorrect diagnosis.

Methods and materials: We collected 4481 frontal Chest X-rays (CXRs), performed in the supine or standing position, labeled by 7 radiologists from the radiology department of Cochin University hospital (AP-HP, Paris,France). Frontal CXR quality criteria included full anatomical coverage, lack of rotation or scapula projection, deep inspiration and optimal exposure. A deep convolutional neural network was trained to classify CXRs as technically correct or incorrect. The model predictions were compared to a ground truth set (15%), labeled by 2 chest radiologist experts.

Results: There were no signifiant differences regarding age, sex, and classification for each criterion between the test, validation and training datasets ($p>0,05$).We first evaluated the inter-rater reliability and found good correlation for each criterion (Cohen's kappa > 0,6). The model performance was evaluated and compared to that of each observer with expert annotation as ground truth. The model performance was close to the radiologists' performance, (accuracies 80-90% compared to 83-95% for radiologists) excepted for the rotation criteria.

Conclusion: The trained model to detect quality-deficient CXRs could be used by technologists in real-time to ensure high-quality images.

Disclosure: N/A

Keywords: Chest X-ray, Quality, Technologists, Artificial intelligence

### SS 11

### The Performance Of AI Covid-19 Detection And Lung Injury Quantification On Chest CT In A Real-time Clinical Setting

*N. Watté, T. Van der Stricht, C. van den Hoven, A. Baetslé, M. van der Meersch, L. Van Hoe, P. Aerts*

Short summary: An artificial intelligence (AI) tool designed to detect COVID-19 on chest CT can be used as a screening tool with high sensitivity but with low specificity. Additional training with supplementary artifact datasets should further improve diagnostic accuracy.

Purpose/objectives: To evaluate the performance of an AI tool, for Covid-19 detection and lung injury quantification, on chest CT during a real-time clinical workflow.

Materials & methods: We retrospectively collected a consecutive dataset of 264 Chest CTs performed to screen for Covid-19 at hospital admission. All axial images were pseudo-anonymized and sent to the AI tool Quibim Precision platform (QUIBIM S.L) to be analyzed by the Imaging COVID-19 Analyzer. The AI tool provided a probability score for COVID-19 infection. RT-PCR was considered the gold standard for COVID-19 diagnosis.

Results: When the COVID-19 probability score cut-off value is set at 0.41 there is a sensitivity of 90.48% (95%CI: 82.09% to 95.80%), specificity of 30.00% (95%CI: 23.42% to 37.26%), PPV of 13.65% (95%CI: 12.32% to 15.11%) and NPV of 96.26 (95%CI: 92.77% to 98.10%) and an AUC of 0.75. Regarding the probabilities we suggest the following ranges with 95% sensitivity to exclude the disease and 95% specificity to include the disease: < 0.38: almost certain negative; 0.39 – 0.62: indeterminate; > 0.63: almost certain positive. We chose a relatively low cut-off value in order to have a high sensitivity so it could be used as a screening test. However, this reduced the specificity and diagnostic accuracy. When using the suggested probability ranges, a substantial number of cases (69%) were labeled as indeterminate. False positive cases were partly explained by mislabeling of breathing artifacts, hypoventilation in dependent lung areas or linear atelectasis as ground glass opacities. Also, some clear-cut diagnoses for the radiologists (e.g., heart failure, bacterial pneumonia, interstitial lung disease, …) were often given a high probability by the AI tool.

Conclusion: The AI tool can be used as a screening tool with a sensitivity of 90% when the cut-off value is set relatively low. Due to low specificity the AI tool on its own cannot be used as a diagnostic test but has the potential to serve as an adjunct for COVID-19 detection. Training with supplementary artifact datasets should further improve the AI accuracy.

Disclosure: Authors have no conflicts of interest to disclose.

Keywords: COVID-19, CT, Artificial Intelligence

### SS 12

### Radiomics analysis enables accurate differential diagnosis between avascular necrosis and transient osteoporosis of the hip

*Michail E. Klontzas, Georgios C. Manikis, Katerina Nikiforaki, Evangelia E. Vassalou, Konstantinos Spanakis, Ioannis Stathis, George A. Kakkos, Nikolas Matthaiou, Aristeidis H. Zibis, Kostas Marias, Apostolos H. Karantanas*

Short Summary: Avascular necrosis and transient osteoporosis of the hip are conditions related to bone marrow edema of the proximal femur. Differentiation between the two entities can be extremely complicated, requiring significant MSK radiology expertise and the combination of imaging and clinical data.

Purpose/Objectives: The aim of this study was to employ radiomics for the differentiation of the two entities with the use of MRI data.

Methods and materials: A total of 109 hips with TOH and 104 hips with AVN were retrospectively included and femoral heads were manually segmented. Radiomics features were extracted with the use of PyRadiomics as implemented in 3D Slicer. Relevant radiomics features (n=38) were selected by removing collinearities and applying Boruta tree-based feature selection. An extreme gradient boosting (XGboost) machine learning model was trained on 70% and validated on 30% of the dataset and the results were compared to the performance of two fellowship-trained MSK radiologists.

Results: XGboost achieved an Area Under the Curve (AUC) of 93.7% (95%CI from 87.7 to 99.8%) whereas the MSK radiologists achieved an AUC of 90.6% (95%CI from 86.7% to 94.5%) and 88.3% (95%CI from 84% to 92.7%) respectively.

Conclusion: In conclusion, radiomics-based machine learning achieved excellent performance, similar to MSK radiologists, in differentiating between TOH and AVN.

Disclosure: Nothing to disclose

Keywords: Radiomics; Artificial Intelligence; avascular necrosis; hip; transient osteoporosis

### SS 13

### Performances of a deep learning algorithm for the detection of fractures, dislocations, elbow joint effusions, focal bone lesions on trauma X-rays

*Jeanne Ventre, Nor-Eddine Regnard, Boubekeur Lanseur, Louis Lassalle, Aurélien Lambert, Benajmin Dallaudière, Antoine Feydy*

Short summary: An artificial intelligence (AI) software that detects skeletal lesions on standard X-rays can help radiologists avoid diagnostic errors.

Purpose/Objectives: To appraise the performances of an AI trained to detect and localize skeletal lesions and compare them to the routine radiological interpretation.

Methods and materials: We retrospectively collected all radiographic examinations with the associated radiologists' reports performed after a traumatic injury during 3 consecutive months (January to March 2017) in a private imaging group of 14 centers. Each examination was analyzed by an AI (BoneView, Gleamer) and its results were compared to those of the radiologists' reports. In case of discrepancy, the examination was reviewed by a senior skeletal radiologist to settle on the presence of fractures, dislocations, elbow effusions, and focal bone lesions (FBL).

Lesion-wise sensitivity, specificity, and NPV of the AI and of the radiologists' report were calculated for each lesion type.

The study received IRB approval n°CRM-2106-177.

Results: A total of 4774 exams were included in the study. Lesion-wise sensitivity was 73.7% for the radiologists' reports vs. 98.1% for the AI (+24.4 points) for fracture detection, 63.3% vs. 89.9% (+26.6 points) for dislocation detection, 84.7% vs. 91.5% (+6.8 points) for elbow effusion detection, and 16.1% vs. 98.1% (+82 points) for FBL detection. The specificity of the radiologists' reports was always 100% whereas AI specificity was 88%, 99.1%, 99.8%, 95.6% for fractures, dislocations, elbow effusions, and FBL respectively. The NPV was measured at 99.5% for fractures, 99.8% for dislocations, and 99.9% for elbow effusions and FBL.

Conclusion: AI has the potential to prevent diagnosis errors by detecting lesions that were initially missed in the radiologists' report. The main limitations are that performance of the AI were calculated stand-alone and that the concordant examinations between the AI and the radiologists' reports were not reviewed by the Ground Truth.

Disclosure: This study was funded by Gleamer

Keywords: Deep learning; fracture; elbow effusion; dislocation; focal bone lesion
